# Sex differences in motor learning flexibility are accompanied by sex differences in mushroom spine pruning of the mouse primary motor cortex during adolescence

**DOI:** 10.3389/fnins.2024.1420309

**Published:** 2024-07-08

**Authors:** Michael Tekin, Hui Shen, Sheryl S. Smith

**Affiliations:** ^1^Department of Physiology and Pharmacology, SUNY Downstate Medical Center, Brooklyn, NY, United States; ^2^Graduate Program in Neural and Behavioral Science, SUNY Downstate Medical Center, Brooklyn, NY, United States; ^3^School of Biomedical Engineering, Tianjin Medical University, Tianjin, China

**Keywords:** GABA_A_ receptor, alpha-4, dendritic spine, motor cortex, sex differences, motor learning, cognitive flexibility

## Abstract

**Background:**

Although males excel at motor tasks requiring strength, females exhibit greater motor learning flexibility. Cognitive flexibility is associated with low baseline mushroom spine densities achieved by pruning which can be triggered by α4βδ GABAA receptors (GABARs); defective synaptic pruning impairs this process.

**Methods:**

We investigated sex differences in adolescent pruning of mushroom spine pruning of layer 5 pyramidal cells of primary motor cortex (L5M1), a site essential for motor learning, using microscopic evaluation of Golgi stained sections. We assessed α4GABAR expression using immunohistochemical and electrophysiological techniques (whole cell patch clamp responses to 100 nM gaboxadol, selective for α4βδ GABARs). We then compared performance of groups with different post-pubertal mushroom spine densities on motor learning (constant speed) and learning flexibility (accelerating speed following constant speed) rotarod tasks.

**Results:**

Mushroom spines in proximal L5M1 of female mice decreased >60% from PND35 (puberty onset) to PND56 (Pubertal: 2.23 ± 0.21 spines/10 μm; post-pubertal: 0.81 ± 0.14 spines/10 μm, *P* < 0.001); male mushroom spine density was unchanged. This was due to greater α4βδ GABAR expression in the female (*P* < 0.0001) because α4 -/- mice did not exhibit mushroom spine pruning. Although motor learning was similar for all groups, only female wild-type mice (low mushroom spine density) learned the accelerating rotarod task after the constant speed task (*P* = 0.006), a measure of motor learning flexibility.

**Conclusions:**

These results suggest that optimal motor learning flexibility of female mice is associated with low baseline levels of post-pubertal mushroom spine density in L5M1 compared to male and female α4 -/- mice.

## Introduction

Sex differences in motor behavior have been reported, where males excel at feats requiring force and speed (Liutsko et al., 2020). However, some studies have suggested that females demonstrate better motor learning flexibility based on more complex and original learning strategies, assessed in humans (Liutsko et al., 2020; Pic et al., 2020) and in teleost fish (Lucon-Xiccato and Bisazza, 2016). These reports suggest that cognitive flexibility may be enhanced in females.

The primary motor cortex (M1) is essential for movement execution (Evarts, 1968; Georgopoulos et al., 1982) as well as motor learning (Kawai et al., 2015). Increases in neuronal activity in M1 accompanies motor learning trials where separate groups of neurons report successful and unsuccessful trials to enable efficient corrective maneuvers (Levy et al., 2020). One well-studied animal task for both short-term and long-term motor skill training is rotarod training (Buitrago et al., 2004). Increases in activity of layer 5 (L5) M1 neurons also accompanies rotarod locomotion learning, while blocking excitatory amino acid receptors in this region impairs learning, pinpointing L5 M1 as a pivotal site for motor learning using the rotarod task (Kida et al., 2023).

Studies have suggested that the learning process results in the transformation of thin (“learning”) spines into mushroom (“memory”) spines (Bourne and Harris, 2007). Indeed, increases in large volume (putatively mushroom) spines are reported after rotarod training in L5 M1 consistent with this theory (Kida et al., 2023). Our previous studies have reported that spatial learning also results in increased density of mushroom spines in CA1 hippocampus, which continue to increase as the spatial target changes (Afroz et al., 2016), suggesting that cognitive flexibility requires additional populations of mushroom spines to emerge, an outcome dependent upon suitable elimination of pre-existing spines (Afroz et al., 2016).

During adolescence, many CNS areas undergo significant loss of spines (Huttenlocher, 1979; Markham et al., 2013; Koss et al., 2014), a process known as “synaptic pruning,” which produces the greatest decreases in the density of the mushroom spines (Afroz et al., 2016; Evrard et al., 2021). This process has relevance for the physiological and behavioral changes occurring at puberty. Puberty is a major developmental transition from childhood to pre-adulthood when reproductive capabilities first emerge. Many of the memories formed pre-pubertally are no longer relevant post-pubertally, and the loss of those synaptic connections via pruning would permit new synaptic connections to be formed which are necessary for optimal behavior post-pubertally into adulthood. Our studies show that in the absence of pruning, mushroom spine density is doubled on dendrites of several CNS areas post-pubertally, including CA1 and CA3 hippocampus as well as the prelimbic (PL) prefrontal cortex (PFC) (Afroz et al., 2016; Parato et al., 2019; Evrard et al., 2021). Under these conditions, cognitive flexibility is impaired (Afroz et al., 2016).

We have shown that adolescent synaptic pruning of CA1 hippocampus is triggered by the emergence of α4βδ GABA_A_ receptors (GABARs) which express on the dendritic spine, adjacent to the excitatory synapse (Shen et al., 2010). Either local or global knock-out of these receptors prevents pruning (Afroz et al., 2016; Evrard et al., 2021), which increases the density of mushroom spines, resulting in impaired spatial learning flexibility (Afroz et al., 2016). These extrasynaptic α4βδ GABARs are activated by ambient GABA and generate a tonic current (Stell and Mody, 2002), which is inhibitory in most areas of the adult CNS (Stell et al., 2003).

In order to investigate potential sex differences in motor learning flexibility, we tested whether there were sex differences in the pruning of mushroom spines in L5 M1 basilar dendrites as we have reported in the PL (Evrard et al., 2021). Although synaptic pruning of L5 M1 apical dendrites has been reported (Tjia et al., 2017), which occurs due to a higher rate of spine elimination than formation, spine type was not assessed. It is not known if synaptic pruning occurs in the basilar dendrites, and importantly, whether the mushroom spine density is decreased during adolescence. In the present study, we also investigated whether pruning of L5 M1 was due to increased expression of α4βδ GABARs at puberty as we have shown is the case in other CNS sites (Afroz et al., 2016). Motor learning flexibility was then assessed on a rotarod learning task. Mice were initially trained to walk on a constant speed rotarod (“motor learning”). The following day, “motor learning flexibility” was assessed as their ability to learn a new task, walking on an accelerating rotarod, testing their ability to re-learn or “update” their locomotor repertoire. We tested the hypothesis that mice with increased mushroom spine density of L5 M1 post-pubertally would show impairments in motor learning flexibility.

## Materials and methods

### Animals

Male and female C57/BL6 mice (“wild-type”, Jackson Labs, Bar Harbor, ME) and α4 -/- mice were housed in a reverse light:dark cycle (12:12). α4 -/- mice were bred on site from α4 +/- mice supplied by G. Homanics (Univ. Pittsburgh). The background strains for α4 -/- mice are C57BL/6J and Strain 129S1/X1. The initial mutation inserted Cre recombinase-activated LoxP (locus of X-over P1) sites flanking exon 3 of the GABRA4 gene (in 129S1/X1 mice) (Chandra et al., 2006). These mice were bred with a Cre expressing mouse strain to delete exon 3 of the GABRA4 gene, and then the Cre was bred out. The α4 gene is transcribed but cannot be translated in these mice because of the frame shift caused by the exon 3 deletion. These mice were back-crossed for 3 generations to C57BL/6J mice, yielding a 99.8% genetically similarity. The mice continue to be back-crossed to C57BL/6J mice every 5 generations. Wild-type mice were used instead of α4 +/+ mice because spine densities in cortex and hippocampus are similar across adolescence (Afroz et al., 2016; Evrard et al., 2021), and thus we routinely combined the data from the 2 strains. In addition, +/+ and wild-type C57BL/6J mice learn equally well to walk on a rotarod and have similar locomotion/activity levels (Chandra et al., 2006). They do not differ electro-physiologically or in terms of GABAR expression (Sabaliauskas et al., 2015). However, we cannot rule out potential unpredictable differences between the two strains. Genotyping of the tails (Transnetyx, Cordova, TN) was used to identify mice that were homozygous α4 -/-.

Mice were tested and euthanized in the dark phase of the light:dark cycle. Mice were tested pre-pubertally (PND 28-31), after puberty onset (female, vaginal opening, ~PND 35; male, ~PND 41) or post-pubertally (PND 56). For PND 56 female mice, estrous cycle stage was assessed by examination of the vaginal cytology; proestrous/estrous mice were not used to avoid possible estrus-associated changes in spine density (Woolley and McEwen, 1992) and activity (Kent et al., 1991). Separate groups of mice were tested for electrophysiological responses (pre-pubertal-PND 28-31, pubertal-PND 35-38), dendritic spine density (pubertal, post-pubertal) and rotarod learning/learning flexibility tasks (post-pubertal, PND 56) during the dark part of the circadian cycle. (Sample sizes: 8 mice/group, spine density; 5 mice/group, immunohistochemistry; 5–6 mice/group, electrophysiology, rotarod test.) Procedures were in accordance with the SUNY Downstate Institutional Animal Care and Use Committee.

### Golgi stain procedure

On the day of brain extraction, mice were first anesthetized with urethane (4 g/kg, i.p. in saline). Brains from pubertal (PND 35-40) and post-pubertal (PND 56) mice were processed using the FD Neurotechnologies Rapid Golgi Stain kit (Columbia, MD) (Afroz et al., 2016). Following a 48 h incubation in Solution C, brains were sliced at 200 μm on a vibratome (Leica VT1200s). Slices were mounted on gelatin-coated slides (FD Neurotechnologies) and cover-slipped.

### Dendritic mushroom spine imaging and analysis

The primary motor cortex was identified by stereotaxic coordinates 0.73 – 2.0 mm anterior to bregma, 1.24–2.15 mm lateral and 0.5–1.1 mm below the pial surface using The Mouse Brain in Stereotaxic Coordinates (4th Edition, Paxinos and Franklin, 2013) and the Allen Brain Institute's Mouse Brain Atlas (http://mouse.brainmap.org). Images of the basilar dendrites of L5M1 pyramidal cells were acquired as Z-stack projection photomicrographs (0.2 μm steps) with a Nikon DS-U3 camera mounted on a Nikon Eclipse Ci-L microscope using a CFI Plan Apochromat DM Lambda 100X oil objective and analyzed with NIS-Elements D 4.40.00 software (Afroz et al., 2016).

Dendrites were classified as proximal (initial 1/3 of the dendrite) or distal (latter 2/3 of the dendrite). Medial and distal regions were merged because they yielded similar spine counts. 2–6 neurons were sampled per mouse, with a maximum of 2 dendrites per neuron. Each dendrite segment was ~1 μm thick and was taken from a 2° or 3° order dendrite. Dendrites were chosen based on having met the following criteria: Unbroken, begins and ends within tissue sample and within the same field of view. They were not obstructed by other dendrites and had a minimum length of 70 μm. Dendrites were also excluded if the surrounding area had a high background or the staining was not clear enough to allow spine characterization for a minimum of 20 μm in the proximal or distal region of the dendrite.

Mushroom spines were counted in each defined region and expressed as number of spines per 10 μm. Head size, neck length, and spine length were evaluated using the “annotation and measurement” tools in NIS-Elements AR. These segments had a length of 20–50 μm within the proximal and distal regions of the dendrite. Segment size was the maximal possible length in this range and excluded areas of dendrite overlap, blurry staining or those areas obstructed by staining artifacts. Mushroom spines were identified as having a head > 0.35 μm and a head:neck > 2 (Arellano et al., 2007; Afroz et al., 2016). This classification has been used previously (Afroz et al., 2016; Evrard et al., 2021). All spine counting was performed with the scientist blinded to the classification of the animal.

### Immunohistochemistry

Mice were anesthetized with urethane (4 g/kg, i.p. in saline), followed by perfusion with saline (12–15 mls/min) and paraformaldehyde [4%, PFA, buffered to pH 7.4 with 0.1 M phosphate buffer (PB)]. Brains were dissected and post-fixed 48 h in 4% PFA at 4°C.

Following sectioning on a vibratome (35 μm coronal sections, Leica VT 100M), free-floating sections were washed (3x) in phosphate buffered saline (PBS)-Tween with 1% bovine serum albumin (BSA) for 10 min. Sections were blocked in PBS supplemented with 1.5% goat serum in PBS-Tween 2 h at room temperature, followed by 2% goat anti-mouse Fab fragments (Jackson Immunolabs, Bar Harbor, ME) for an additional 2 h. Then, sections were incubated with anti-α4 (mouse monoclonal, Antibodies, Inc., Davis, CA, 1:100) overnight at 4°C. This antibody is selective for detection of the GABAR α4 subunit (Evrard et al., 2021). After washing, sections were incubated with Alexa Fluor 594 (1:1,000) for 2 h the following day. Following a final washing, sections were mounted on slides with ProLong Glass antifade reagent with 5% DAPI (4′,6-diamidino-2-phenylindole) a blue-emitting fluorescent compound used for nuclear staining (ThermoFisher Scientific). Images were taken with an Olympus FluoView TM FV1000 confocal inverted microscope with objective UPLSAPO 40 × or 100 × NA:1:30 (Olympus, Tokyo, Japan). Layer 5 M1 was identified by its anatomical coordinates (0.5–1.1 mm below the pial surface) and the presence of larger somata than for layers 2/3 and 6. For the immunohistochemical analysis, the merged z-stack image (2 μm steps) was used. ROIs were analyzed for image luminosity (fluorescence intensity) in the original image using Adobe Photoshop after subtracting the adjacent background levels.

### Electrophysiology

Cortical slice preparation. Brains from euthanized pre-pubertal and pubertal female mice were removed and cooled using an ice-cold solution of artificial cerebrospinal fluid (aCSF) containing (in mM): NaCl 124, KCl 2.5, CaCl_2_ 2, NaH_2_PO_4_ 1.25, MgSO_4_ 2, NaHCO_3_ 26, and glucose 10, saturated with 95% O2, 5% CO2, (pH, 7.4). Brains were sectioned at 400 μm on a Leica VT1000S vibratome and incubated for 1 h in oxygenated aCSF.

Cortical slice voltage-clamp electrophysiology. Pyramidal cells in L5 M1 were visualized using a differential interference contrast (DIC)-infrared upright Leica microscope and recorded using whole-cell patch clamp procedures in voltage clamp mode at 26–30°C (Evrard et al., 2021).

Patch pipets were fabricated from borosilicate glass using a Flaming-Brown puller to produce open tip resistances of 2–4 MΩ. For recordings of the tonic inhibitory current, the pipet solution contained in mM: CsCl 140, HEPES 5, EGTA 5, CaCl_2_-H_2_O 0.5, QX-314 5, Mg-ATP 2, Li-GTP 0.5, pH 7.2, 290 mOsm. 5 mM QX-314 was added to block voltage-gated Na+ channels and GABA_B_ receptor-activated K+ channels. The aCSF contained 50 μM kynurenic acid which blocks AMPA and NMDA receptors, as well as 0.5 μM TTX to isolate the post-synaptic component. Recordings were carried out at a −60 mV holding potential, and the tonic current was assessed by the change in holding current in response to 100 nM gaboxadol, a GABAR agonist which, at this concentration, is selective for δ-containing GABAR (Brown et al., 2002; Jia et al., 2005; Meera et al., 2011). The GABAergic nature of the current was verified by block with gabazine (GBZ, SR95531, 120 μM). Drugs were bath applied continuously in sequential order following 5–10 min of baseline recordings without drugs. Recordings were conducted with a 2 kHz 4-pole Bessel filter at a 10 kHz sampling frequency using an Axopatch 200B amplifier and pClamp 9.2 software. Electrode capacitance and series resistance were monitored and compensated; access resistance was monitored throughout the experiment, and cells were discarded if the access resistance increased more than 10% during the experiment. In all cases, the data represent one recording/animal.

### Rotarod learning experiments

In order to test motor learning and motor learning flexibility in mouse groups with high proximal mushroom spine density (male wild-type, female α4-/-) and low proximal mushroom spine density (female wild-type) post-pubertally, mice were trained to walk on a rotarod (Rota-Rod R/S LE8500C from Panlab, Harvard Apparatus), using the latency to fall across 4 consecutive trials as a measure of learning. On day one, the mice were habituated to the rotarod for 2 runs at 0 RPM. On day two, after performing a control trial at 0 RPM, motor learning was tested across four 5-min trials at a constant rotarod speed of 4 RPM. The difference between the latency to fall on trial 1 vs. trial 4 was considered a measure of motor learning. (If trial 3 yielded a longer latency to fall, this number was used instead.) These numbers were compared in individual mice to see if learning occurred, and then group averages (difference in the latency to fall across trial 1 vs. 4) compared across groups to see if there was a difference in motor learning ability.

On day 3, motor learning flexibility was tested across four 5-min trials at an accelerating speed of 4 to 40 RPM. Motor learning flexibility was analyzed in the same way as motor learning, described above. On each day, the mice were habituated to the room for 1 hour before interacting with the rotarod, and there was a 30 min break between each trial. In some cases, male wild-type and female α4-/- mice were tested directly on the accelerating rotarod without prior training on the constant speed rotarod, as a measure of learning rather than learning flexibility.

### Statistics

In the Results and Tables, the mean, standard error of the mean (S.E.M.) and 95% confidence intervals are presented. In figures showing spine density, the mean, median, 25–75% range, interquartile range and individual data points are shown. For the behavior graphs, the mean and S.E.M. are presented as well as the individual data points. In most cases, statistical analyses were performed with OriginLab software (2023 version). For spine density assessments where the data were normally distributed (assessed using the Kolmogorov-Smirnoff test), comparisons between two groups were made using a nested t-test (Prism, GraphPad). In cases where the spine density data were not normally distributed (mushroom spine counts for pubertal and post-pubertal males and females), a generalized mixed linear model was constructed; the dependent variable was mushroom spine count, distributed as an over-dispersed Poisson variable. Log of distance was used as an offset variable. Fixed factors were sex, age group and their interaction; animal ID was introduced as a random factor. Residual variance was estimated separately for each sex; Kenward-Roger adjustments to standard errors and denominator degrees of freedom were applied. Model residuals were inspected for outliers, and no outliers were detected. Model-generated means & 95% confidence intervals (CIs) are presented. *Post-hoc* analysis of sex differences in pubertal and post-pubertal mushroom spine counts on the proximal dendrites was accomplished using the non-parametric two-sample Kolmogorov-Smirnoff test.

The immunohistochemical and electrophysiology data were assessed using the student's *t* test. Individual latency to fall data for motor learning (constant speed rotarod) did not follow a normal distribution. Therefore, individual comparisons between trial 1 and trial 4 for each group were made with a non-parametric Wilcoxon signed ranks test. Individual latency to fall data for motor learning flexibility (accelerating rotarod) followed a normal distribution. Therefore, these comparisons were made with a paired *t* test. For both motor learning and motor learning flexibility, learning data were distributed normally, and comparisons between groups were conducted using an Analysis of Variance with a *post-hoc* Fisher's test. In all cases, a *P* < 0.05 was used to signify statistical significance.

## Results

### Sex differences in proximal mushroom spine pruning of L5 M1 of the female wild-type mouse

**Figure 1:**
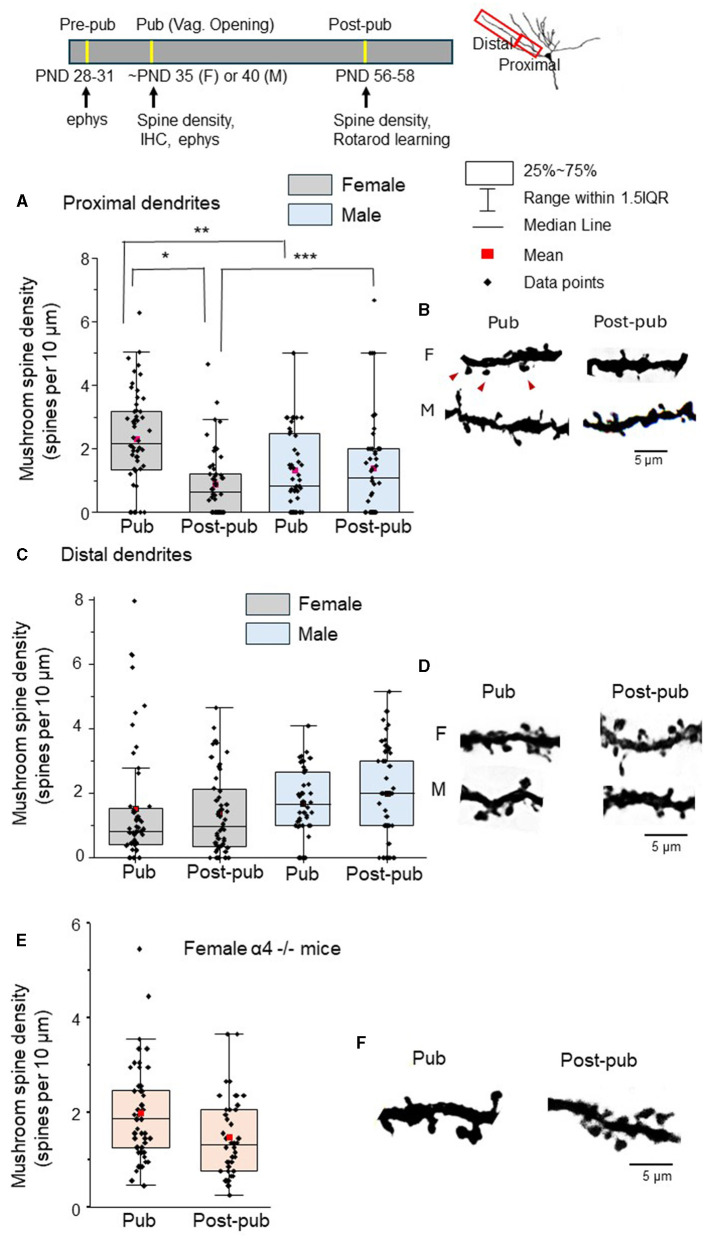
Mushroom spine density of L5 M1 proximal dendrites decreases during adolescence only in wild-type female mice. Insets, timeline of ages used for testing (ephys, electrophysiology; IHC, immunohistochemistry); diagram of ranges of dendrites used for spine density analysis; the scatter plot indicates the mean (red), median, 25-75% range, the 1.5 interquartile range and the individual data points for each group. **(A)** Mushroom spine density (spines/10 μm) on the proximal dendrites for female (left) and male (right) pubertal (Pub) and post-pubertal (Post-pub, PD 56) mice. There was a significant age by sex interaction (*P* < 0.001) assessed using a generalized mixed linear model. Mushroom spine density decreased significantly across adolescence only in female mice (^*^P < 0.001) but not for males (*P* = 0.767) determined using simple effects analysis Mushroom spine density was greater for pubertal females vs. pubertal males (^**^P = 0.00083) while mushroom spine density was lower for post-pubertal females vs. post-pubertal males (^***^P = 0.03). (spine counts, *n* = 4–12 dendrites from 8 mice/group). **(B)** Representative images. F, female; M, male; red arrows, mushroom spines. **(C)** Scatter plot of the mushroom spine density (spines/10 μm) on the distal dendrites for female (left) and male (right) pubertal (Pub) and post-pubertal (Post-pub, PD 56) mice. Using a generalized mixed linear statistical model, there was no significant age by sex interaction (*P* = 0.590). In a model without the interaction term, there were no significant age (*P* = 0.916) or sex (*P* = 0.330) main effects (spine counts, *n* = 4–10 dendrites from 8 mice/group). **(D)** Representative images. **(E)** Scatter plot of the mushroom spine density (spines/10 μm) on the proximal dendrites of pubertal (Pub) and post-pubertal (Post-pub, PND 56) female α4 -/- mice. There was no significant change in spine density [t_(14)_ = 1.45, *P* = 0.17, spine counts, *n* = 4–6 dendrites from 8 mice/group]. **(F)** Representative images.

Our previous studies have revealed that the predominant spine-type which undergoes pruning in CA1 hippocampus (Afroz et al., 2016) and prelimbic prefrontal cortex (Evrard et al., 2021) during adolescence is the mushroom spine, thought to represent “memory” (Bourne and Harris, 2007). Thus, we tested whether mushroom spine pruning would also be evident in L5 M1 at puberty, comparing spine density in male and female mice at the onset of puberty and post-pubertally. Spine density in the female proximal L5M1 dendrites decreased by >60% during adolescence (Figures 1A, B, Table 1, Pub: 2.23 ± 0.21 spines/10 μm vs. Post-pub: 0.81 ± 0.14 spines/10 μm), in contrast to the male data, which did not reveal a difference (Figures 1A, B, Table 1, Pub: 1.28 ± 0.31 spines/10 μm vs. Post-pub: 1.36 ± 0.36 spines/10 μm). There was a significant age by sex interaction [*F*_(1, 46)_=13.33, *P* < 0.001]. Simple effects analysis showed a significant age effect for females [*F*_(1, 39)_ = 23.78, *P* < 0.001] but not for males [*F*_(1, 40)_=0.09, *P* = 0.767], suggesting that there are sex differences for mushroom spine pruning on the proximal L5M1 dendrites. *Post-hoc* analysis (2 sample Kolmogorov-Smirnoff test) revealed significantly more mushroom spines for the female at puberty compared to the male (female, 2.23 ± 0.21 spines/10 μm vs. male, 1.28 ± 0.31 spines/10 μm, D = 0.38, z=1.92, *P* = 0.00083). In addition, there were 42% fewer mushroom spines on the proximal dendrites of the female mouse post-pubertally compared to the male (female, 0.81 ± 0.14 spines/10 μm vs. male, 1.36 ± 0.36 spines/10 μm, D = 0.274, z = 1.37, *P* = 0.036).

**Table 1:** Means and upper and lower limits of the 95% confidence intervals (CI) for mushroom spine density in layer 5 primary motor cortex (proximal and distal dendrites) for males and females according to pubertal status (pubertal, Pub; post-pubertal, post-pub).

**Area**	**Sex**	**Pub status**	**Mean**	**Lower 95% CI**	**Upper 95% CI**
Proximal	Female	Pub	2.32	1.77	2.82
		Post-pub	0.81	0.62	1.19
	Male	Pub	1.29	0.98	1.69
		Post-pub	1.37	1.02	1.83
Distal	Female	Pub	1.57	0.97	2.56
		Post-pub	1.41	0.86	2.31
	Male	Pub	1.75	1.09	2.80
		Post-pub	2.03	1.25	3.28

In contrast, spine density of distal mushroom spines in L5 M1 did not change from puberty to post-puberty in either females or males (Figures 1C, D, Table 1, female, Pub: 1.52 ± 0.26 spines/10 μm vs. post-pub: 1.41 ± 0.18 spines/10 μm; male, Pub: 1.04 ± 0.15 spines/10 μm vs. Post-pub: 1.41 ± 0.20 spines/10 μm). There was no significant age by sex interaction [*F*_(1, 27)_=0.30, *P* = 0.590]. In a model without the interaction term, there was no significant age [*F*_(1, 28)_=0.01, *P* = 0.916] or sex [*F*_(1, 28)_=0.98, *P* = 0.330] main effect. These findings suggest that mushroom spine pruning is only observed in the female proximal L5 M1 during adolescence.

Our previous findings suggest that α4βδ GABARs trigger adolescent pruning of mushroom spines in the CA1 hippocampus and prelimbic prefrontal cortex because α4 -/- mice do not undergo mushroom spine pruning at puberty (Afroz et al., 2016; Evrard et al., 2021). Therefore, we tested the hypothesis that these receptors would also play a role in pruning of mushroom spines in female L5M1 such that pruning would not be observed in the α4 -/- mouse. Indeed, the density of mushroom spines did not change significantly [t_(14)_ = 1.45, *P* = 0.17] between puberty and post-puberty (Figures 1E, F, Table 2, Pub: 1.9 ± 0.14 spines/10 μm vs. post-pub: 1.5 ± 0.13 spines/10 μm) in L5M1 of the female α4 -/-. These data suggest that α4βδ GABARs trigger pruning of mushroom spines in the female L5M1 as we have shown in other CNS areas.

**Table 2:** Means and upper and lower limits of the 95% confidence intervals (CI) for proximal mushroom spine density in layer 5 primary motor cortex for female α4-/- mice according to pubertal status (pubertal, Pub; post-pubertal, Post-pub).

**Pubertal status**	**Mean**	**Lower 95% CI**	**Upper 95% CI**
Pub	1.96	1.68	2.25
Post-pub	1.51	1.24	1.77

Pubertal expression of the GABAR α4 subunit in is greater in female L5 M1.

**Figure 2:**
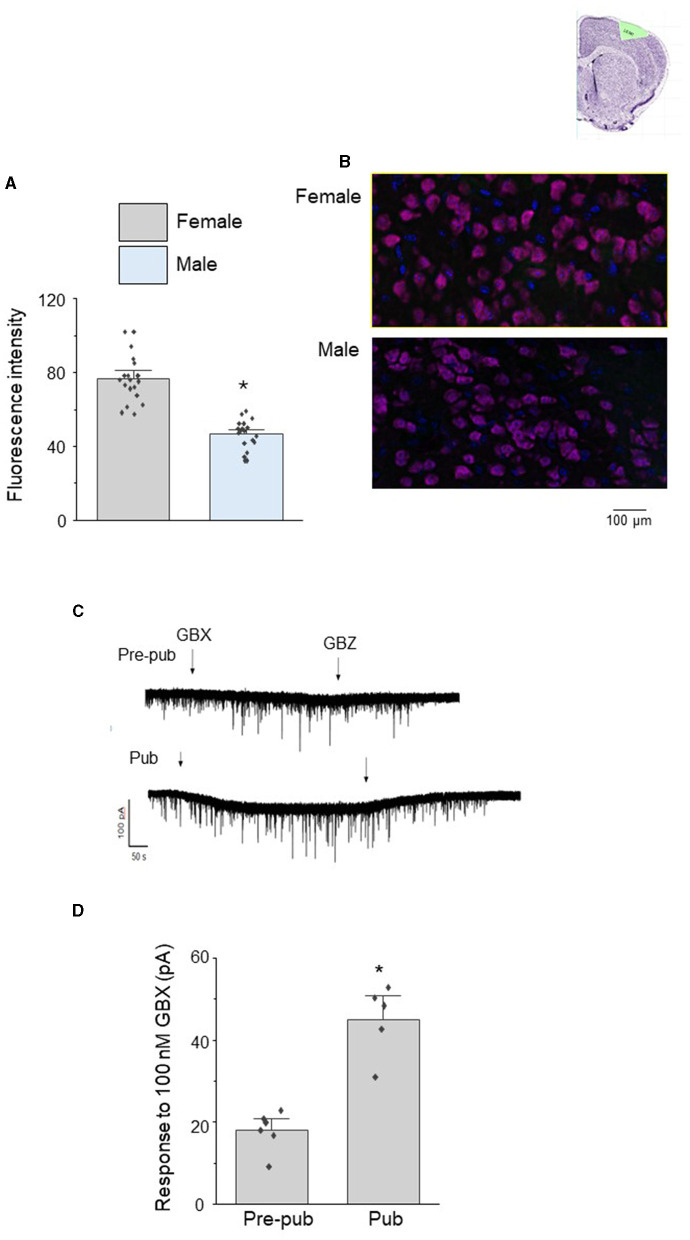
Sex differences in the pubertal expression of the GABAR α4 subunit in L5 M1 pyramidal cells. **(A)** Averaged data (mean ± S.E.M.) of α4 fluorescence units from pubertal male and female mice assessed using immunohistochemical techniques, t_(19)_ = 9.45, *P < 0.0001 vs. female (*n* = 4 cells/mouse, 5 mice/group). **(B)** Representative images. α4, magenta; DAPI, blue. **(C)** Representative whole cell voltage clamp recordings of L5 M1 pyramidal cell responses to the GABAR agonist gaboxadol (GBX, 100 nM, arrow 1) in slices from pre-pubertal (pre-pub) and pubertal (Pub) female mice. This concentration of gaboxadol is selective for α4βδ GABARs, and the change in the holding current in response to GBX application is a measure of functional α4βδ GABAR expression. GBZ, gabazine (SR95531, 120 μM, arrow 2), a GABAR antagonist. **(D)** Averaged data, mean ± S.E.M., t_(8)_ = 5.8, *P < 0.0002 vs. pre-pub. (*n* = 5–6 recordings/group, 1 recording/mouse).

We tested whether sex differences in the pubertal expression of α4βδ GABARs could underlie the observed sex differences in pubertal pruning of mushroom spines in L5 M1. To this end, we examined pubertal expression of the GABAR α4 subunit using immunohistochemical techniques. Expression of α4 in female L5 M1 was 65% greater than that of the male, assessed by the fluorescence intensity (Figures 2A, B, female, 76.8 ± 2.9, 74.9 and 83.8, 95% confidence intervals; male, 46.5 ± 1.8, 46.0 and 55.1, 95% confidence intervals; t_(19)_=9.45, *P* = 1.2 x 10^−8^).

In order to assess whether the pubertal increase in α4βδ GABAR expression in female L5 M1 represented functional expression, we recorded the holding current response of L5M1 pyramidal cells to local application of 100 nM gaboxadol, a GABA agonist which is selective for α4βδ GABARs at this concentration (Jia et al., 2005; Meera et al., 2011). Indeed, the response to gaboxadol increased by ~150% at puberty compared to pre-puberty (Figures 2C, D, Pre-pub: 19.5 ± 1.5 pA vs. Pub: 47 ± 4 pA; t_(8)_=5.8, *P* < 0.0002) as revealed by the change in the holding current assessed using whole cell patch clamp techniques in the slice preparation. This gaboxadol-generated current was blocked completely by 120 μm gabazine (Figure 2C), reflecting the GABAergic nature of the current. These results suggest that functional expression of α4βδ GABARs increases at puberty in female L5M1 where they generate a tonic current.

Increases in proximal mushroom spine density of L5M1 post-pubertally are associated with impairments in motor learning flexibility.

**Figure 3:**
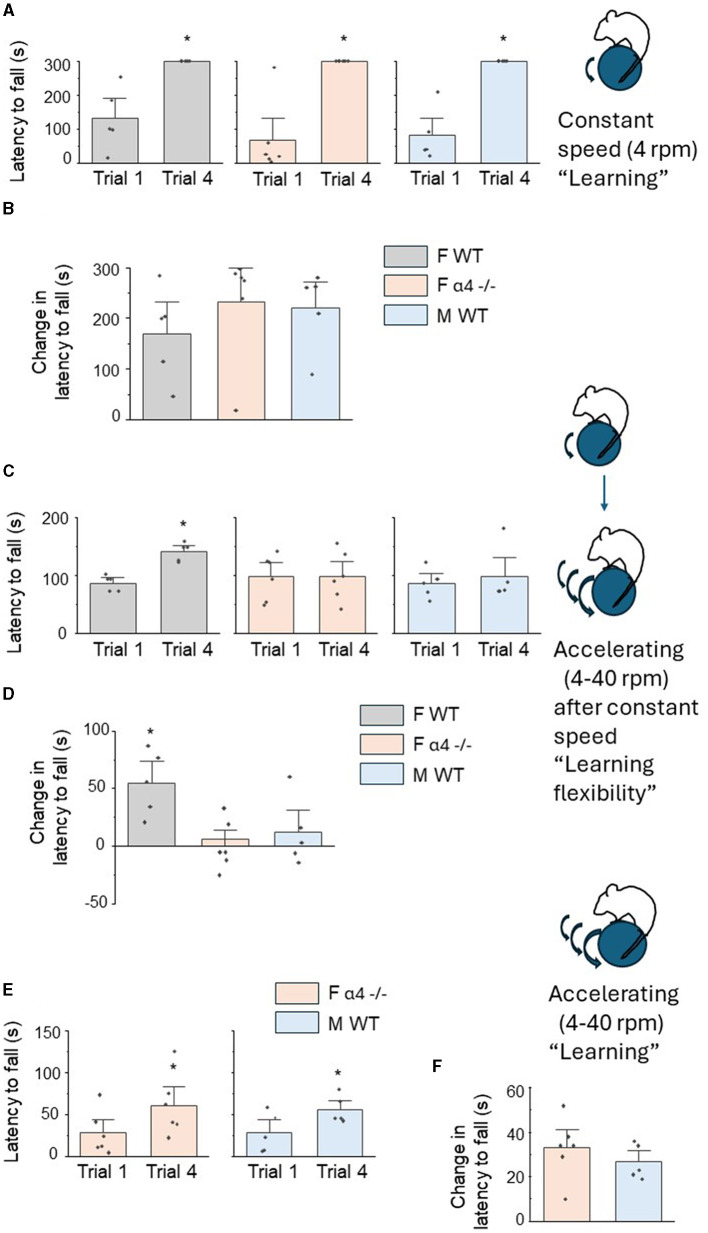
Sex differences in motor learning flexibility on a rotarod, but not motor learning, are associated with differences in proximal mushroom spine density of L5 M1. **(A)** Inset, diagram of constant speed rotarod task. Post-pubertal (PND 56) mice were tested on a constant speed (4 RPM for 5 min) rotarod across 4 trials. In this and the following figures, the mean ± S.E.M are presented. Scatter plots of latency to fall for trial 1 vs. trial 4 for female wild-type (F WT, left), female α4 -/- (F α4 -/-) and male wild-type (M WT). All comparisons were significantly different F WT, t_(4)_ = 4.2, *P = 0.007, F α4 -/-, t_(5)_ = 5.3, *P = 0.002, M WT, t_(4)_ = 6.4, *P = 0.002, *n* = 5–6 mice/group. **(B)** Averaged data for the change in the latency to fall (T4 – T1), a measure of learning, for all groups. There were no significant differences between groups [F_(2, 13)_ = 0.66, *P* = 0.53]. *n* = 5–6 mice/group. **(C)** Inset, diagram of motor learning flexibility. Mice were tested on an accelerating (4–40 RPM for 5 min) rotarod across 4 trials the day following initial constant speed training. Scatter plots of latency to fall for trial 1 vs. trial 4 for female wild-type (F WT, left), female α4 -/- (F α4 -/-) and male wild-type (M WT) mice. Only F WT mice demonstrated a significant learning effect for this altered learning protocol (“re-learning”), F WT, t_(4)_ = 4.4, *P = 0.006, F α4 -/-, t_(5)_ = 0.10, *P* = 0.46, M WT, t_(4)_ = 0.90, *P* = 0.21, *n* = 5–6 mice/group. **(D)** Averaged data for the change in the latency to fall (T4 – T1). There was a significant increase in the change in the latency to fall from T1 to T4 for F WT compared to the other groups [F_(2, 13)_ = 6.40, *P* = 0.012]. *P < 0.05 vs. other groups, *n* = 5–6 mice/group. **(E)** Inset, diagram of accelerating rotarod learning with no prior experience. Scatter plots of latency to fall for trial 1 vs. trial 4 for female α4 -/- (F α4 -/-) and male wild-type (M WT) mice. Both groups demonstrated a significant learning effect, F α4 -/-, t_(5)_ = 5.9, *P = 0.001, M WT, t_(4)_ = 6.9, *P = 0.0012, *n* = 5–6 mice/group. **(F)** Averaged data for the change in the latency to fall (T4 – T1). There were no significant differences between groups [t_(9)_ = 0.90, *P* = 0.39], *n* = 5–6 mice/group.

Mice were initially tested for motor learning on a constant speed rotarod (4 RPM) across 4 learning trials. In order to assess learning for each group, latency to fall on trial 4 was compared to latency to fall on the first trial. Using these criteria, each group significantly improved performance across the four 5-min trials (*P* < 0.05) ultimately maintaining position on the rotarod for the full 5 min (Figure 3A, Supplementary Figures 1A–C). Latency to fall was not significantly different for the initial trial [F_(2, 13)_ = 0.66, *P* = 0.53], suggesting that innate ability was not different between groups. The change in latency to fall across trials, a measure of learning, was also not different between the groups [Figure 3B, female wildtype: 170 ± 40.8 s, female α4 -/-: 233 ± 43.7 s, male wild-type: 220 ± 34.6 s, F_(2, 13)_ = 0.66, *P* = 0.53], suggesting that differences in proximal mushroom spine density of L5M1 do not impact learning a motor task.

Our previous findings suggest that increased mushroom spine density of CA1 hippocampus resulting from knock-out of α4βδ GABARs impairs cognitive flexibility on hippocampal-specific spatial learning tasks (Afroz et al., 2016). For this study, we tested the hypothesis that increases in mushroom spine density post-pubertally due to a lack of proximal dendrite pruning of L5M1 in male wild-type and female α4 -/- mice during adolescence would impair motor learning flexibility on a rotarod task compared to the female wild-type which undergoes pruning of mushroom spines. Motor learning flexibility was tested as the ability to adapt to a new rotarod protocol (accelerating rotarod) after having learned a previous protocol (constant speed rotarod). This task is dependent upon M1 activity (Kida et al., 2023).

Motor learning flexibility was tested across groups, assessing the animals' performance on a rotarod with an accelerating rotation (4–40 RPM over the 5 min period) for 4 learning trials. Again, learning was assessed comparing the latency to fall on trial 4 with trial 1. In this case, only the female wild-type mice significantly improved performance [Figure 3C, T1: 87.4 ± 6.1 s, T4: 142 ± 7.0 s, t_(4)_ = 4.4, *P* = 0.0116, Supplementary Figure 1D], compared to the other two groups [Figure 3C, female α4 -/-, T1: 98.3 ± 15.7 s, T4: 99.2 ± 17.1 s, t_(5)_ = 0.10, *P* = 0.93, male wild-type:, T1: 86.8 ± 11.1 s, T4: 98.6 ± 21.3 s, t_(4)_ = 0.90, *P* = 0.42, Supplementary Figures 1E, F]. These data suggest that female wild-type mice alone displayed motor learning flexibility. This was also reflected in the change in the latency to fall (T4 vs. T1), a measure of learning where the performance of the female wild-type was significantly better than the other two groups [Figure 3D, female wildtype: 55 ± 412.5 s, female α4 KO: 0.83 ± 8.7 s, male wild-type: 11.8 ± 13.0 s, F_(2, 13)_ = 6.4, *P* = 0.0116]. However, as observed for the learning experiment, latency to fall during the initial trial on the accelerated rotarod was not significantly different between the groups [female wildtype: 87.4 ± 6.1 s, female α4 KO: 98.3 ± 15.8 s, male wild-type: 86.8 ± 11.1 s, F_(2, 13)_ = 0.29, *P* = 0.75], again suggesting that innate ability did not differ between groups. These results demonstrate that only the female wild-type mouse can successfully learn (i.e., improve performance on) a novel motor task in four trials, reflecting optimal motor learning flexibility. Because this group was the only one to undergo pruning of mushroom spines on the proximal dendrites of L5M1 to result in fewer mushroom spine post-pubertally, the data also suggest an inverse association of mushroom spine density with motor learning flexibility.

We also tested whether the groups (male wild-type, female α4 KO) with higher post-pubertal mushroom spine density on the proximal L5M1 dendrites could improve performance on the accelerating rotarod task when it was implemented as a learning task rather than a learning flexibility task. To this end, performance on the accelerating rotarod task was tested without prior training at a constant speed. In both cases, latency to fall from T1 to T4 significantly increased, reflecting learning [Figure 3E, female α4 -/-: T1: 28.5 ± 10.5 s, T4: 61.3 ± 15.0 s, t_(5)_ = 5.9, *P* = 0.001, male wild-type: T1: 28.8 ± 10.5 s, T4: 56.2 ± 7.2 s, t_(4)_ = 6.9, *P* = 0.0012, Supplementary Figures 1G, H]. The change in latency to fall was not different between groups [Figure 3F, female α4 KO: 32.8 ± 5.6 s, male wild-type: 26.6 ± 3.5 s, t_(9)_ = 0.9, *P* = 0.39]. These results suggest that learning (improvement of performance) can be observed for post-pubertal male wild-type and female α4 KO mice on the accelerating rotarod task when it represents the initial motor learning task.

## Discussion

The results from this study suggest that sex differences in the pruning of mushroom spines in M1 during adolescence which enable a lower mushroom spine density post-pubertally are associated with sex differences in motor learning flexibility. Mushroom spine pruning in L5 M1 of the pubertal female mouse was dependent upon the emergence of α4βδ GABARs, as we have shown for other CNS areas (Afroz et al., 2016), because mushroom pruning was not observed after α4 knock-out. Pubertal α4 expression was greater in female L5 M1, which may underlie the observed sex differences in mushroom spine pruning. Higher mushroom spine densities in both post-pubertal male wild-type and female α4 -/- mice were associated with impaired motor learning flexibility compared to the post-pubertal female wild-type mouse suggesting that optimal motor learning flexibility is associated with a low baseline mushroom spine density. Although not tested, it would be expected for male α4 -/- mice to have higher spine densities than the wild-type males resulting in further impairments in motor learning flexibility.

M1 encodes the speed, direction and force of limb movement with laminar stratification of forelimb representations (Evarts, 1968; Georgopoulos et al., 1982; Moran and Schwartz, 1999). The M1 is also a critical center for motor skill learning as shown by the fact that lesions of M1 prevent learning a new motor skill (Kawai et al., 2015). The learning process involves both L3 M1 which reports motor performance (success vs. failure) and L5 activity which monitors the consequences of the previous action and uses this information to correct ongoing movements and to inform future movements (Levy et al., 2020). Increased activity of M1 neurons accompanies rotarod learning, while local blockade of AMPA or NMDA receptors with CNQX or APV, respectively, impairs rotarod learning confirming rotarod training as a learning process. Large volume (mushroom) spine density is increased following rotarod training (Li et al., 2017; Kida et al., 2023), as is EPSC frequency/amplitude and the AMPAR/NMDAR ratio, consistent with increases in potentiated synapses which would result from motor learning (Kida et al., 2023). Although the M1 is the predominant site responsible for learning the rotarod task, we cannot rule out effects of other CNS areas which project to M1which may account for the observed sex differences in motor learning flexibility. These include premotor, executive and sensory centers, as well as the ventrolateral thalamus (Bedwell et al., 2014) which has high levels of α4 expression (Jia et al., 2005). However, determining selective sites responsible for the changes in learning flexibility would require a region- and layer-specific knock-out.

In the present study, motor learning flexibility was tested by administering a new accelerating rotarod protocol after a previous constant speed protocol was successfully learned. Post-pubertal wild-type female mice, who have a significantly lower (~42%) density of mushroom spines on the proximal dendrites of L5 M1 pyramidal cells compared to the male, learned the new task, as assessed by an increase in latency to fall by the fourth trial compared to the first trial. Neither the male wild-type nor the female α4 -/- mice, who do not undergo pruning and have higher levels of mushroom spines on the proximal dendrites post-pubertally, improved performance over the 4 training trials. This suggests an impairment in learning flexibility although these mice might show improved performance with additional trials. However, when tested without prior constant speed training, both male wild-type and female α4 -/- mice displayed significant improvement (learning) from trial 1 to trial 4 on the accelerated rotarod task, suggesting that impairments in motor learning flexibility were not due to intrinsic deficits in sensorimotor behavior. In addition, the fact that there were similar performance scores for the first trial supports this conclusion as well as the fact that all three groups performed equally well on the learning task.

As suggested by our previous experimental work (Afroz et al., 2016) as well as by theoretical analysis (Chechik et al., 1999), having an appropriately low baseline mushroom spine density may facilitate re-learning new protocols (i.e., learning flexibility). In our studies in CA1 hippocampus (Afroz et al., 2016), we assessed mushroom spine density during hippocampal-dependent spatial learning and re-learning (i.e., cognitive flexibility) in post-pubertal (PND 56) female mice. Each learning episode resulted in more mushroom spines on the CA1 pyramidal cell dendrites. In mice which did not prune (α4 -/- mice), the pre-existing mushroom spine density was significantly higher than wild-type at the start of the experiment (as in the present study). Although the first learning episode increased the mushroom spines in the α4 -/-, the second learning episode did not. This was associated with impaired cognitive flexibility of the α4 -/- mice on the hippocampal learning task. Because both initial learning and subsequent learning (flexibility) processes increase mushroom spine density, a higher mushroom spine density at baseline may preclude the additional increases following re-learning due to spatial constraints or to energy requirements. Similar changes in spine plasticity are likely to have occurred in the present study during the rotarod learning and learning flexibility tasks based on studies which show that rotarod training increases mushroom spines (Li et al., 2017; Kida et al., 2023).

Other papers have reported similar findings to the present study (Kovacs and Pearce, 2013; Hernandez et al., 2020) showing superior performance of females on an accelerating rotarod but others report no sex difference in accelerating rotarod performance (Chari et al., 2020; Tsao et al., 2023). Reasons for this discrepancy include an effect of diet which has been reported (Kovacs and Pearce, 2013), drum diameter (Shiotsuki et al., 2010) or the light:dark cycle (Chari et al., 2020), some of which could possibly affect α4 gene expression and alter dendritic spine density. We tested mice during the dark, while Chari et al. (2020) tested during the light phase of the cycle which may explain the discrepancies.

The fact that both groups with higher mushroom spine density displayed impaired motor learning flexibility argues against this being due to some other sex difference in motor ability because female α4 -/- mice performed similarly to wild-type male mice. Numerous studies have shown that α4 -/- mice have normal sensori-motor activity where open field activity, time on a constant speed rotarod and pain threshold are not altered (Chandra et al., 2006, 2008) nor is anxiety-like behavior in the δ-/- mouse (Shen et al., 2007). The female mice used in the present rotarod learning study were excluded if they were in the proestrous/estrous (P/E) stage of the ovarian cycle. This would result in estrous stages (diestrus, metestrus) that are associated with similar levels of locomotor activity (assessed by the running wheel) compared to males (Ferguson et al., 2021).

Our previous findings have revealed that mushroom spines are the predominant, or in some cases, the only spine type pruned during adolescence in a number of brain areas, including CA1 hippocampus, CA3 hippocampus and PL PFC (Afroz et al., 2016; Parato et al., 2019; Evrard et al., 2021). Similar sex differences are found in L3 and L5 PFC, where only females undergo adolescent pruning (Markham et al., 2013; Evrard et al., 2021). It is thought that pubertal synaptic pruning removes underused synaptic contacts to make room for new synapses as learning continues in adulthood. Indeed, many brain areas in both humans and rodents have been shown to undergo significant levels (~50%) of spine removal during adolescence (Huttenlocher, 1979; Zehr et al., 2006; Markham et al., 2013; Koss et al., 2014; Pattwell et al., 2016). There are multiple factors which play a role in pruning. Our studies have shown that the emergence of α4βδ GABARs which localize on dendritic shafts and spines, close to the excitatory synapse (Shen et al., 2010), are the trigger for pruning in some areas (Afroz et al., 2016; Evrard et al., 2021). These receptors have a high sensitivity to ambient GABA, maintained by the GABA transporters, and little desensitization, permitting them to generate a tonic inhibitory current (Bianchi et al., 2002; Brown et al., 2002; Stell and Mody, 2002). α4βδ receptors impair the activation of NMDA receptors on the spine which in turn reduces the expression of kalirin-7 (Kal7) (Afroz et al., 2016). Kal7 is a Rho guanine nucleotide exchange factor (Rho-Gef) which is necessary for spine maintenance (Ma et al., 2008; Penzes and Jones, 2008). Global knock-out of α4βδ GABARs prevents pruning during adolescence as does local knock-down.

The sex difference in pubertal mushroom spine pruning of L5 M1 is also likely due to the sex difference in pubertal α4 expression. Female α4 expression was significantly greater than that of the male at puberty. These were shown to be functional receptors based on the increased responses of pyramidal cells to gaboxadol which are selective for α4βδ GABARs. However, increases in excitatory drive to male M1 during puberty may also contribute to the lack of pruning here. During adolescence, the male M1 develops dense populations of androgen receptors (Kritzer, 2004) and testosterone produces more sustained motor evoked potentials than observed in the female (Pitcher et al., 2003). This increased activity may override the inhibition generated by the α4βδ GABARs and result in reduced spine loss. However, we cannot rule out the possibility that mushroom spine pruning occurs at an earlier age of development in the male wild-type and/or female α4 -/- mice due to other mechanisms.

Many studies report that males have greater ability to execute gross motor tasks requiring increased muscular force compared to females (Liutsko et al., 2020). However, several studies suggest that females excel at motor learning flexibility, that is, learning a new motor repertoire appropriate for novel conditions (Sjoberg and Cole, 2018; Pic et al., 2020). The motor response to the start signal of a GO/NO GO task was found to be similar in males and females; however, females performed significantly better for the less frequent stop signal (Sjoberg and Cole, 2018), suggesting a greater ability to learn the new response. Differences in reaction time and visual field effects were both ruled out. A similar trend was reported for a manual aiming task, where both sexes performed equally well on the initial task, but females did better when the criteria changed (Fernandes et al., 2017). Response to distractors was not different between the sexes, suggesting that sex differences in cognitive flexibility were likely responsible for the results. Similar findings have been reported in teleost fish (Lucon-Xiccato and Bisazza, 2016).

The results from the present study reveal sex differences in adolescent pruning of mushroom spines in proximal L5M1 due to the emergence of α4βδ GABARs at puberty. Male wild-type mice and female α4 -/- mice, with higher post-pubertal mushroom spine densities in L5 M1, did not perform as well on a rotarod acceleration re-learning task, suggesting that an appropriately low baseline mushroom spine density may improve motor learning flexibility. These results may be relevant for autism spectrum disorder (ASD) where impairments in motor learning and cognitive flexibility are reported (Gidley Larson and Mostofsky, 2008; Memari et al., 2014) in association with defective synaptic pruning (Hutsler and Zhang, 2010; Van Eylen et al., 2011; Tang et al., 2014). Sex differences in cognitive flexibility have been reported in ASD (Demetriou et al., 2021). ASD is also associated with abnormal variants of GABRA4 (Ma et al., 2005). Thus, the results from the present study may be relevant for the motor impairments reported for this disorder.

## Data availability statement

The raw data supporting the conclusions of this article will be made available by the authors, without undue reservation.

## Ethics statement

The animal study was approved by Institutional Animal Care and Use Committee of SUNY Downstate Medical Center. The study was conducted in accordance with the local legislation and institutional requirements.

## Author contributions

MT: Data curation, Formal analysis, Investigation, Visualization, Writing – original draft, Writing – review & editing. HS: Data curation, Formal analysis, Investigation, Writing – review & editing. SS: Conceptualization, Formal analysis, Funding acquisition, Project administration, Supervision, Writing – original draft, Writing – review & editing.

## References

[B1] Afroz S. Parato J. Shen H. Smith S. S. (2016). Synaptic pruning in the female hippocampus is triggered at puberty by extrasynaptic GABAA receptors on dendritic spines. Elife. 5:e043. 10.7554/eLife.15106.043 PMC4871702 27136678

[B2] Arellano J. I. Benavides-Piccione R. Defelipe J. Yuste R. (2007). Ultrastructure of dendritic spines: correlation between synaptic and spine morphologies. Front Neurosci. 1:2007. 10.3389/neuro.01.1.1.010.2007 18982124 PMC2518053

[B3] Bedwell S. A. Billett E. E. Crofts J. J. Tinsley C. J. (2014). The topology of connections between rat prefrontal, motor and sensory cortices. Front. Syst. Neurosci. 8:177. 10.3389/fnsys.2014.00177 25278850 PMC4166227

[B4] Bianchi M. T. Haas K. F. Macdonald R. L. (2002). Alpha1 and alpha6 subunits specify distinct desensitization, deactivation and neurosteroid modulation of GABA (A) receptors containing the delta subunit. Neuropharm 43, 492–502. 10.1016/S0028-3908(02)00163-6 12367596

[B5] Bourne J. Harris K. M. (2007). Do thin spines learn to be mushroom spines that remember? Curr.Opin.Neurobiol. 17, 381–386. 10.1016/j.conb.2007.04.009 17498943

[B6] Brown N. Kerby J. Bonnert T. P. Whiting P. J. Wafford K. A. (2002). Pharmacological characterization of a novel cell line expressing human alpha (4)beta (3)delta GABA (A) receptors. Br. J. Pharmacol. 136, 965–974. 10.1038/sj.bjp.0704795 12145096 PMC1573424

[B7] Buitrago M. M. Schulz J. B. Dichgans J. Luft A. R. (2004). Short and long-term motor skill learning in an accelerated rotarod training paradigm. Neurobiol Learn Mem. 81, 211–216. 10.1016/j.nlm.2004.01.001 15082022

[B8] Chandra D. Jia F. Liang J. Peng Z. Suryanarayanan A. Werner D. F.. (2006). GABA-A receptor alpha-4 subunits mediate extrasynaptic inhibition in thalamus and dentate gyrus and the action of gaboxadol. Proc Natl Acad Sci. 103, 15230–15235. 10.1073/pnas.0604304103 17005728 PMC1578762

[B9] Chandra D. Werner D. F. Liang J. Suryanarayanan A. Harrison N. L. Spigelman I.. (2008). Normal acute behavioral responses to moderate/high dose ethanol in GABA-A receptor alpha4 subunit knock-out mice. Alc Clin Exp Res. 32, 10–18. 10.1111/j.1530-0277.2007.00563.x 18076749 PMC2896280

[B10] Chari T. Griswold S. Andrews N. A. Fagiolini M. (2020). The stage of the estrus cycle is critical for interpretation of female mouse social interaction behavior. Front. Behav. Neurosci. 14:113. 10.3389/fnbeh.2020.00113 32714163 PMC7340104

[B11] Chechik G. Meilijson I. Ruppin E. (1999). Neuronal regulation: a mechanism for synaptic pruning during brain maturation. Neural Comp. 11, 2061–2080. 10.1162/089976699300016089 10578044

[B12] Demetriou E. A. Pepper K. L. Park S. H. Pellicano L. Song Y. J. C. Naismith S. L.. (2021). Autism spectrum disorder: an examination of sex differences in neuropsychological and self-report measures of executive and non-executive cognitive function. Autism. 25, 2223–2237. 10.1177/13623613211014991 34169770

[B13] Evarts E. V. (1968). Relation of pyramidal tract activity to force exerted during voluntary movement. J Neurophysiol. 31, 14–27. 10.1152/jn.1968.31.1.14 4966614

[B14] Evrard M. R. Li M. Shen H. Smith S. S. (2021). Preventing adolescent synaptic pruning in mouse prelimbic cortex via local knockdown of alpha4betadelta GABAA receptors increases anxiety response in adulthood. Sci Rep. 11:21059. 10.1038/s41598-021-99965-8 34702942 PMC8548505

[B15] Ferguson L. Giza C. C. Serpa R. O. Greco T. Robert H. Folkerts M.. (2021). Sex differences in neurophysiological changes following voluntary exercise in adolescent rats. Front. Neurol. 12, 685822. 10.3389/fneur.2021.685822 34367052 PMC8339288

[B16] Fernandes L. A. Ugrinowisch H. Ventura de Oliveira J. R. Salvador M. G. Antunes Bicalho L. E. Lage G. M. (2017). Comparison between manual aiming control and sex in different task constraints. Motriz 23, 16. 10.1590/S1980-6574201700030029

[B17] Georgopoulos A. P. Kalaska J. F. Caminiti R. Massey J. T. (1982). On the relations between the direction of two-dimensional arm movements and cell discharge in primate motor cortex. J Neurosci. 2, 1527–1537. 10.1523/JNEUROSCI.02-11-01527.1982 7143039 PMC6564361

[B18] Gidley Larson J. C. Mostofsky S. H. (2008). Evidence that the pattern of visuomotor sequence learning is altered in children with autism. Autism Res. 1, 341–353. 10.1002/aur.54 19360689 PMC2892291

[B19] Hernandez A. R. Truckenbrod L. M. Campos K. T. Williams S. A. Burke S. N. (2020). Sex differences in age-related impairments vary across cognitive and physical assessments in rats. Behav. Neurosci. 134, 69–81. 10.1037/bne0000352 31886694 PMC7078049

[B20] Hutsler J. J. Zhang H. (2010). Increased dendritic spine densities on cortical projection neurons in autism spectrum disorders. Brain Res. 1309, 83–94. 10.1016/j.brainres.2009.09.120 19896929

[B21] Huttenlocher P. R. (1979). Synaptic density in human frontal cortex - developmental changes and effects of aging. Brain Res. 163, 195–205. 10.1016/0006-8993(79)90349-4 427544

[B22] Jia F. Pignataro L. Schofield C. M. Yue M. Harrison N. L. Goldstein P. A. (2005). An extrasynaptic GABAA receptor mediates tonic inhibition in thalamic VB neurons. J. Neurophysiol. 94, 4491–4501. 10.1152/jn.00421.2005 16162835

[B23] Kawai R. Markman T. Poddar R. Ko R. Fantana A. L. Dhawale A. K.. (2015). Motor cortex is required for learning but not for executing a motor skill. Neuron 86, 800–812. 10.1016/j.neuron.2015.03.024 25892304 PMC5939934

[B24] Kent S. Hurd M. Satinoff E. (1991). Interactions between body temperature and wheel running over the estrous cycle in rats. Physiol. Behav. 49, 1079–1084. 10.1016/0031-9384(91)90334-K 1896490

[B25] Kida H. Kawakami R. Sakai K. Otaku H. Imamura K. Han T.-Z.. (2023). Motor training promotes both synaptic and intrinsic plasticity of layer V pyramidal neurons in the primary motor cortex. J. Physiol. 601, 335–353. 10.1113/JP283755 36515167

[B26] Koss W. A. Belden C. E. Hristov A. D. Juraska J. M. (2014). Dendritic remodeling in the adolescent medial prefrontal cortex and the basolateral amygdala of male and female rats. Synapse 68, 61–72. 10.1002/syn.21716 24105875

[B27] Kovacs A. D. Pearce D. A. (2013). Location- and sex-specific differences in weight and motor coordination in two commonly used mouse strains. Sci Rep. 3:2116. 10.1038/srep02116 23817037 PMC3698490

[B28] Kritzer M. (2004). The distribution of immunoreactivity for intracellular androgen receptors in the cerebral cortex of hormonally intact adult male and female rats: localization in pyramidal neurons making corticocortical connections. Cereb Cortex. 14, 268–280. 10.1093/cercor/bhg127 14754867

[B29] Levy S. Lavzin M. Benisty H. Ghanayim A. Dubin U. Achvat S.. (2020). Cell-type-specific outcome representation in the primary motor cortex. Neuron. 107, 954–971 e9. 10.1016/j.neuron.2020.06.006 32589878

[B30] Li W. Ma L. Yang G. Gan W. B. (2017). REM sleep selectively prunes and maintains new synapses in development and learning. Nat Neurosci. 20, 427–437. 10.1038/nn.4479 28092659 PMC5535798

[B31] Liutsko L. Muiños R. Ral J. M. T. Contreras M. J. (2020). Fine motor precision tasks: sex differences in performance with and without visual guidance across different age groups. Behav. Sci. 10:36. 10.3390/bs10010036 31963114 PMC7017231

[B32] Lucon-Xiccato T. Bisazza A. (2016). Male and female guppies differ in speed but not in accuracy in visual discrimination learning. Anim Cogn. 19, 733–744. 10.1007/s10071-016-0969-2 26920920

[B33] Ma D. Q. Whitehead P. L. Menold M. M. Martin E. R. Ashley-Koch A. E. Mei H.. (2005). Identification of significant association and gene-gene interaction of GABA receptor subunit genes in autism. Am. J. Hum.Genet. 77, 377–388. 10.1086/433195 16080114 PMC1226204

[B34] Ma X. M. Kiraly D. D. Gaier E. D. Wang Y. Kim E. J. Levine E. S.. (2008). Kalirin-7 is required for synaptic structure and function. J Neurosci. 28, 12368–12382. 10.1523/JNEUROSCI.4269-08.2008 19020030 PMC2586970

[B35] Markham J. A. Mullins S. E. Koenig J. I. (2013). Periadolescent maturation of the prefrontal cortex is sex-specific and is disrupted by prenatal stress. J. Comp. Neurol. 521, 1828–1843. 10.1002/cne.23262 23172080 PMC4479145

[B36] Meera P. M W. Otis T. (2011). Molecular basis for the high THIP/gaboxadol sensitivity of extrasynaptic GABA-A receptors. J.Neurophysiology. 106, 2057–2011. 10.1152/jn.00450.2011 21795619 PMC3191842

[B37] Memari A. H. Ghanouni P. Shayestehfar M. Ziaee V. Moshayedi P. (2014). Effects of visual search vs. auditory tasks on postural control in children with autism spectrum disorder. Gait Posture. 39, 229–234. 10.1016/j.gaitpost.2013.07.012 23931847

[B38] Moran D. W. Schwartz A. B. (1999). Motor cortical representation of speed and direction during reaching. J Neurophysiol. 82, 2676–2692. 10.1152/jn.1999.82.5.2676 10561437

[B39] Parato J. Shen H. Smith S. S. (2019). alpha4betadelta GABAA receptors trigger synaptic pruning and reduce dendritic length of female mouse CA3 hippocampal pyramidal cells at puberty. Neuroscience 398, 23–36. 10.1016/j.neuroscience.2018.11.032 30496825 PMC6411036

[B40] Pattwell S. S. Liston C. Jing D. Ninan I. Yang R. R. Witztum J.. (2016). Dynamic changes in neural circuitry during adolescence are associated with persistent attenuation of fear memories. Nat Commun. 7:11475. 10.1038/ncomms11475 27215672 PMC4890178

[B41] Paxinos G. Franklin K. B. J. (2013). The Mouse Brain in Stereotaxic Coordinates, 4th Edn. New York, NY: Academic Press; Elsevier.

[B42] Penzes P. Jones K. A. (2008). Dendritic spine dynamics–a key role for kalirin-7. Trends Neurosci. 31, 419–427. 10.1016/j.tins.2008.06.001 18597863 PMC3973420

[B43] Pic M. Navarro-Adelantado V. Jonsson G. K. (2020). Gender differences in strategic behavior in a triadic persecution motor game identified through an observational methodology. Front Psychol. 11:109. 10.3389/fpsyg.2020.00109 32116919 PMC7011959

[B44] Pitcher J. B. Ogston K. M. Miles T. S. (2003). Age and sex differences in human motor cortex input-output characteristics. J. Physiol. 546, 605–613. 10.1113/jphysiol.2002.029454 12527746 PMC2342521

[B45] Sabaliauskas N. Shen H. Molla J. Gong Q. H. Kuver A. Aoki C.. (2015). Neurosteroid effects at alpha4betadelta GABAA receptors alter spatial learning and synaptic plasticity in CA1 hippocampus across the estrous cycle of the mouse. Brain Res. 1621, 170–86. 10.1016/j.brainres.2014.12.026 25542386 PMC4480201

[B46] Shen H. Gong Q. H. Aoki C. Yuan M. Ruderman Y. Dattilo M.. (2007). Reversal of neurosteroid effects at alpha4-beta2-delta GABA-A receptors triggers anxiety at puberty. Nat Neurosci. 10, 469–477. 10.1038/nn1868 17351635 PMC1858651

[B47] Shen H. Sabaliauskas N. Sherpa A. Fenton A. A. Stelzer A. Aoki C.. (2010). A critical role for GABA-A receptors in shaping learning deficits at puberty in mice. Science 327, 1515–1518. 10.1126/science.1184245 20299596 PMC2887350

[B48] Shiotsuki H. Yoshimi K. Shimo Y. Funayama M. Takamatsu Y. Ikeda K.. (2010). A rotarod test for evaluation of motor skill learning. J. Neurosci. Methods 189, 180–185. 10.1016/j.jneumeth.2010.03.026 20359499

[B49] Sjoberg E. A. Cole G. G. (2018). Sex differences on the go/no-go test of inhibition. Arch. Sex Behav. 47, 537–542. 10.1007/s10508-017-1010-9 28608292

[B50] Stell B. M. Brickley S. G. Tang C. Y. Farrant M. Mody I. (2003). Neuroactive steroids reduce neuronal excitability by selectively enhancing tonic inhibition mediated by delta subunit-containing GABA-A receptors. Proc. Natl. Acad. Sci. 100, 14439–14444. 10.1073/pnas.2435457100 14623958 PMC283610

[B51] Stell B. M. Mody I. (2002). Receptors with different affinities mediate phasic and tonic GABA (A) conductances in hippocampal neurons. J. Neurosci. 22:RC223. 10.1523/JNEUROSCI.22-10-j0003.2002 12006605 PMC6757628

[B52] Tang G. Gudsnuk K. Kuo S.-H. Cotrina M. L. Rosoklija G. Sosunov A.. (2014). Loss of mTOR-dependent macroautophagy causes autistic-like synaptic pruning deficits. Neuron 83, 1131–1143. 10.1016/j.neuron.2014.07.040 25155956 PMC4159743

[B53] Tjia M. Yu X. Jammu L. S. Lu J. Zuo Y. (2017). Pyramidal neurons in different cortical layers exhibit distinct dynamics and plasticity of apical dendritic spines. Front. Neural Circuits 11:43. 10.3389/fncir.2017.00043 28674487 PMC5474458

[B54] Tsao C.-H. Wu K.-Y. Su N. C. Edwards A. Huang G.-J. (2023). The influence of sex difference on behavior and adult hippocampal neurogenesis in C57BL/6 mice. Sci. Rep. 13:17297. 10.1038/s41598-023-44360-8 37828065 PMC10570284

[B55] Van Eylen L. Boets B. Steyaert J. Evers K. Wagemans J. Noens I. (2011). Cognitive flexibility in autism spectrum disorder: explaining the inconsistencies? Res. Autism Spect. Disord. 5, 1390–1401. 10.1016/j.rasd.2011.01.02523220737

[B56] Woolley C. S. McEwen B. S. (1992). Estradiol mediates fluctuation in hippocampal synapse density during the estrous cycle in the adult rat. J. Neurosci. 12, 2549–2554. 1613547 10.1523/JNEUROSCI.12-07-02549.1992 PMC6575846

[B57] Zehr J. L. Todd B. J Schulz K. M. McCarthy M. M. Sisk C. L. (2006). Dendritic pruning of the medial amygdala during pubertal development of the male Syrian hamster. J. Neurobiol. 66, 578–590. 10.1002/neu.20251 16555234

