# Supplementary figures and images for: Sex differences in motor learning flexibility are accompanied by sex differences in mushroom spine pruning of the mouse primary motor cortex during adolescence
Source: Front Neurosci. 2024 Jul 8;18:1420309. doi: 10.3389/fnins.2024.1420309 (PMC11262054; doi:10.3389/fnins.2024.1420309)

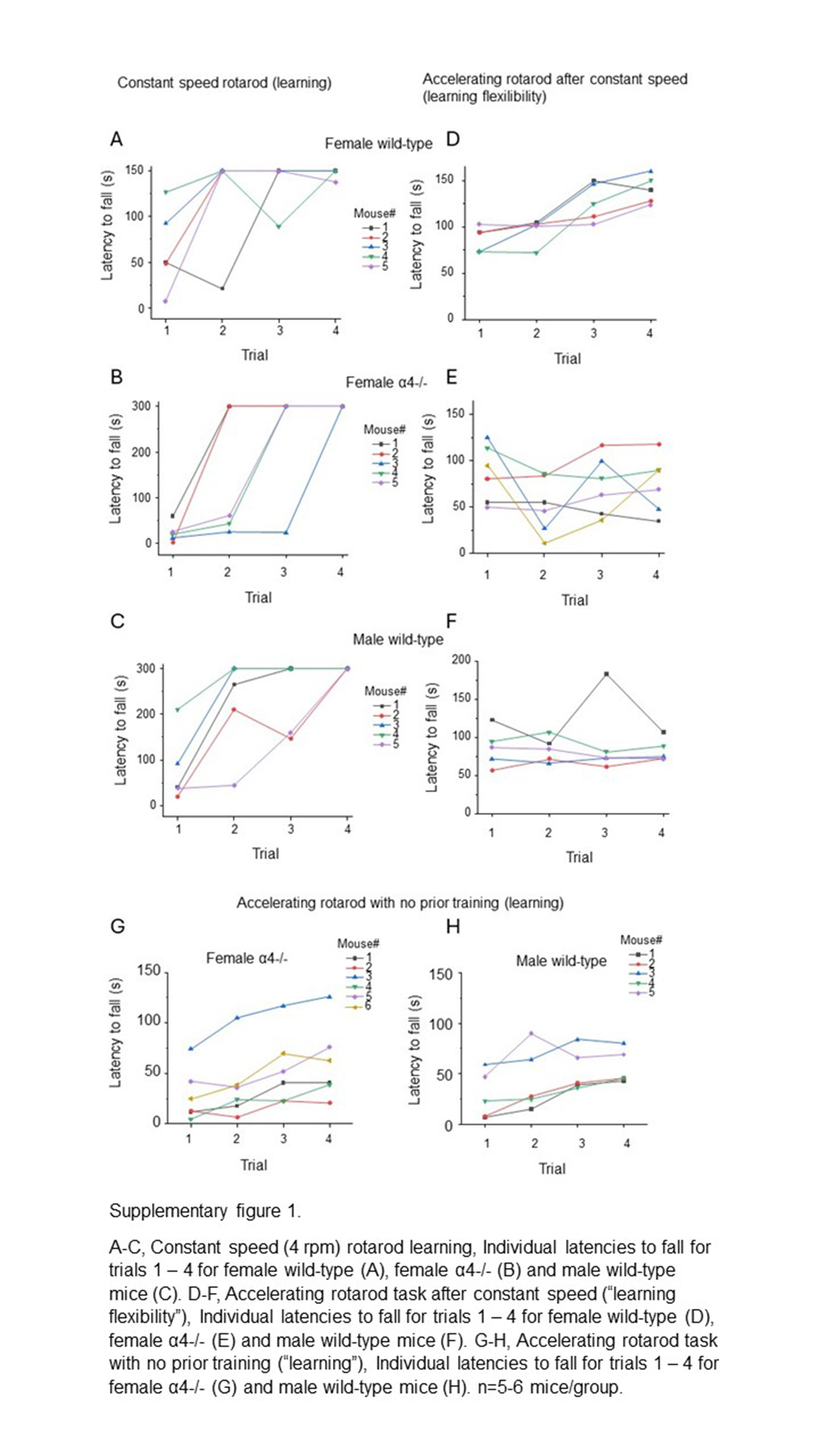

Supplement: Supplementary file 1 [file Image_1.JPEG]
